# The terminal density reversal phenomenon of aging human red blood cells

**DOI:** 10.3389/fphys.2013.00171

**Published:** 2013-07-09

**Authors:** Virgilio L. Lew, Teresa Tiffert

**Affiliations:** Department of Physiology, Development and Neuroscience, University of CambridgeCambridge, UK

That red blood cells (RBCs) become progressively denser with age has become a universally acknowledged truth. However, as with all universally acknowledged truths, troublesome singularities often arise. We focus here on one particular departure from the rule, the terminal density reversal (TDR) phenomenon, discovered by Bookchin (Bookchin et al., 2000), and documented so far in RBCs from healthy subjects (Bookchin et al., 2000; Lew et al., 2007), from sickle cell anemia patients (SS RBCs) (Bookchin et al., 2000; Franco et al., 2000; Lew et al., 2002; Holtzclaw et al., 2002), from patients with β-thalassemia intermedia (Bookchin et al., 2000), and from diabetic subjects with sustained high levels of glycated hemoglobin, Hb A1c (Bookchin et al., 2009).

There are a number of reasons that justify focusing on TDR: it is of substantial physiological and pathophysiological relevance as it defines the late and terminal homeostatic condition of RBCs in the circulation both in health and disease; it remains controversial and in need of further independent confirmation; its mechanism is still poorly understood, and on the evidence so far it is not yet clear whether it is the common final path of all RBCs or of only a selected RBC subpopulation.

Let us start with a brief review of the facts. TDR was first reported by Bookchin et al. (2000). The original observations were made while studying the time-dependent changes in the volume distribution patterns of K^+^-permeabilized RBCs suspended in plasma-like media, as they became progressively dehydrated by the net loss of KCl and water. Bookchin's surprising discovery was that small fractions of cells (0.03 to 4%) failed to dehydrate, both in RBC samples from normal subjects (about 0.05%) and from patients with sickle cell Anaemia (about 4%). Because of certain additional clues he speculated that these dehydration-resistant cells could represent a pre-terminal condition of RBCs in the circulation. Scepticism by his co-worker (Virgilio L. Lew), who suspected an artifactual origin of the results, led to the extended investigation that finally confirmed Bookchin's original findings and supported his interpretations. A brief explanation as to why these findings were so surprising follows below.

The two most widely used procedures to selectively increase the K^+^ permeability of RBCs in suspension are addition of suitable concentrations of the K^+^ ionophore valinomycin or of a divalent cation ionophore, A23187 or ionomycin, in the presence of Ca^2+^ in the medium. Valinomycin acts directly as the K^+^ pathway whereas the divalent cation ionophore requires down-gradient Ca^2+^ influx in excess of the powerful Ca^2+^-extrusion capacity of the plasma membrane calcium pump (PMCA), to build up [Ca^2+^]_i_ to activating levels for the endogenous Ca^2+^-sensitive K^+^ channel of the RBC plasma membrane (kcnn4, Gardos channel). Extensive data from many laboratories had shown that when RBCs are suspended in isotonic, low-K media, both these procedures cause rapid dehydration of the cells by the net loss of KCl and water, driven by the outwardly directed electrochemical gradient of K^+^, and rate-limited by the anion permeability of the cells.

So the failure of some cells to dehydrate was against all previous experience. After ruling out exotic possibilities such as a low anion permeability or ionophore-resistance, the only explanation left was that dehydration-resistance had to result from unexpected sodium gains balancing potassium loses thus preventing dehydration and densification of the cells in low-K media. But how? Scepticism, rooted on experience, was based on one of the side-effects of ionophore addition, not generally reported. Ionophore incorporation in RBCs membranes always causes haemolysis of a small fraction of cells, intensified when the cells are additionally Ca^2+^-loaded (Tiffert et al., 1984). It seemed plausible then that small proportions of pre-haemolytic cells had become leaky to cations, gained sodium and thus resisted dehydration during the procedures originally applied by Bookchin.

So the first and most important test was to establish whether the dehydration-resistant cells had gained sodium *in vivo* or *in vitro*. This was resolved in a series of experiments aimed at preventing any possible sodium gains *in vitro* by repeatedly washing RBCs from freshly extracted blood with large volumes of sodium-free isotonic media before testing for dehydration resistance. The sodium and potassium content of untreated RBCs, and of ionophore-treated RBCs, both dehydrated and dehydration-resistant, was measured by atomic absorption spectroscopy in samples from sickle cell anaemia patients and by X-ray microanalysis in single cells from healthy subjects. In all instances, dehydration-resistant cells exhibited reversed Na/K ratios relative to whole blood controls and to ionophore-dehydrated cells, particularly dramatic in RBCs from normal subjects where the Na/K ratio of dehydration-resistant cells was 10 to 18 fold higher than that in the dehydrated cells (Bookchin et al., 2000). These results established that the high sodium content of the dehydration-resistant cells had been gained *in vivo* in the circulation, and that there were real red cell subpopulations whose intravascular condition was of high-Na, low-K, the vast majority of RBCs. Ancillary measurements by Minetti and collaborators (Minetti et al., 2001) showed that in freshly drawn normal RBCs the increase in sodium content with cell density appeared to be gradual reflecting either progressive net sodium gain with cell age or the redistribution pattern of the high-sodium cells among the density fractions, or both.

The next questions about these dehydration-resistant cells concerned their physiological and pathophysiological significance, and the mechanisms by which they become high-Na, low-K in the circulation. Bookchin's original suspicion that these cells represented a terminal circulatory condition was based on them being found highly enriched among the lightest, reticulocyte-rich fraction of SS RBCs, and with a shape reminiscent of that of the hyperdense irreversible sickled cells (ISCs), as if towards their demise from the circulation they had become sodium-loaded, light and thus dehydration-resistant following K^+^-permeabilization. All these initial results were confirmed by additional studies on SS RBCs which also revealed a highly increased pump-leak sodium-potassium traffic across the membrane of the dehydration-resistant cells, with an unexpected bumetanide-sensitive flux-component (Bookchin et al., 2000; Holtzclaw et al., 2002).

In an elegant series of experiments, Franco and collaborators (Franco et al., 2000; Holtzclaw et al., 2002) labelled and re-infused dense SS RBCs into the original donors and followed their appearance among the light cell fractions, unambiguously demonstrating their formation from denser cells. They showed that it was possible to rehydrate dense sickle cells *in vitro* by continuous oxygenation and by rapid oxy/deoxy cycling, that the *in vitro* rehydrated cells were sodium loaded and potassium depleted, that bumetanide inhibited rehydration in oxy conditions, and, most importantly, that the presence of calcium in the medium was an absolute requirement for rehydration.

This calcium dependence opened the possibility of investigating TDR in normal RBCs from healthy subjects, which was much more difficult than in SS RBCs because of their minute proportion. Using the fraction of glycated hemoglobin, Hb A1c, as a reliable measure of RBC age, and newly developed methodologies to separate cells with different calcium pumping capacity (PMCA *V*_max_) (Lew et al., 2003, 2007), we were able to define the modality of PMCA *V*_max_ decline with RBC age and also separate a small fraction of high-Na and high-Hb A1c RBCs, which resisted dehydration when exposed to ionophore A23187 and calcium. These results demonstrated that, as with SS RBCs, the subpopulation of high-Na normal RBCs represented old RBCs near the end of their circulatory life span.

The next question, as to how aging RBCs cells become Na^+^-loaded and K^+^-depleted *in vivo* by Ca^2+^-dependent processes, remains open. The high permeability of the dehydration-resistant SS RBCs documented in the early studies suggested an age-dependent activation of a poorly-selective cation permeability pathway, tentatively named Pcat, and challenging suggestions about its possible molecular nature have recently been advanced (Thomas et al., 2011).

The following is presented as a tentative working hypothesis on the mechanism of TDR cell formation based on the available evidence. As RBCs age, PMCA *V*max declines sharply (Lew et al., 2007). The consequent progressive increase in [Ca^2+^]_i_ was estimated to be within the 20 to 100 nM range, too low to be detectable by calcium measurements *ex vivo* (Tiffert and Lew, 1997), yet sufficient to increase the time-averaged open-state probability of the Gardos channels (Grygorczyk, 1987), leading to a sustained net loss of KCl and water, with consequent cell densification, the dominant trend for most of the RBC life span. At some stage along this process, near the end of the cell's circulatory life, calpain proteolysis stimulated by the sustained high [Ca^2+^]_i_ levels, or increased membrane protein phosphorylation resulting from macromolecular crowding within the dense cells (Ciana et al., 2004), or some other mechanism yet to be identified, trigger Pcat activation initiating net NaCl gain. This reduces the dehydration rate led by net KCl loss and prevents RBC densification to levels incompatible with normal circulatory flow thus extending the viable RBC life span. Eventually, as the K^+^ gradient dissipates, the homeostatic balance progresses to a stage in which net NaCl gain exceeds net KCl loss beyond the restorative capacity of the sodium pump, causing cell rehydration and terminal density reversal in some or all RBCs.

All the evidence discussed thus far points to TDR as a real phenomenon. However, Lutz and collaborators (Alaia et al., 2009) provided data hard to reconcile with TDR being the general terminal homeostatic path of all RBCs. Using a direct antiglobulin test (DAT) for IgG, they examined density fractionated normal RBCs to detect those with elevated numbers of IgG molecules on their surface. Bound IgG not only contained anti-band 3, but also anti-idiotypic and anti-C3 naturally occurring antibodies. The latter two suppress complement-dependent RBC phagocytosis and were thus expected to prolong RBC life span. They found that IgG-DAT-positive blood donors carried most IgG molecules on dense RBCs and had more RBCs of high density than DAT-negative controls. Their densest RBCs were older than the oldest RBCs of DAT-negative controls, based on the band 4.1a/b ratio, an alternative RBC age measure. They argued that if re-swelling leading to TDR had occurred *in vivo* to a significant extent, it would be hard to explain the presence of the overaged RBCs in the high density fractions documented in their experiments. They speculated that TDR may be the result of mechanical-oxidative damage, known to occur in sickle cells, and also during RBC preparatory washing procedures. But perhaps the critical words here are “significant extent,” and TDR is the final path of only a subpopulation of RBCs, hard to detect among other dominant density subpopulations.

In conclusion, although the evidence for the *in vivo* presence of aged, high-Na, low-density RBCs and SS RBCs seems unassailable, TDR remains controversial and in need of further study.
